# NLRP3 Inflammasome in Stress-Related Neuropsychiatric Disorders: Mechanisms of Neuron–Microglia–Astrocyte Crosstalk, HPA Axis Dysregulation, and Therapeutic Perspective

**DOI:** 10.3390/biom15091344

**Published:** 2025-09-19

**Authors:** Izabela Woźny-Rasała, Ewa Alicja Ogłodek

**Affiliations:** Collegium Medicum, Jan Dlugosz University in Częstochowa, Waszyngtona 4/8 Street, 42-200 Częstochowa, Poland; e.oglodek@ujd.edu.pl

**Keywords:** caspase-1, interleukin-1β, interleukin-18, neuroimmunology, NLRP3 inflammasome, stress, proinflammatory cytokines, stress-related disorders

## Abstract

Chronic stress disrupts neuroimmune homeostasis and initiates CNS inflammation. This paper examines the molecular and cellular mechanisms that connect stress to the interplay among the nervous, endocrine, and immune systems, with a focus on the role of the NLRP3 inflammasome in neuroinflammatory processes. It discusses the dynamics of HPA axis, stress-induced changes in glucocorticoid and mineralocorticoid signaling, sympathetic nervous system activation, and the contribution of pro-inflammatory cytokines in brain immune activation. The NLRP3 inflammasome is described in terms of its structure, activation via a two-signal model, and its role in IL-1β and IL-18 maturation in neurons, microglia, and astrocytes. Preclinical evidence highlights the therapeutic potential of targeting NLRP3 in stress-related disorders, underscoring its key role in their pathophysiology.

## 1. Introduction

**Figure 1:**
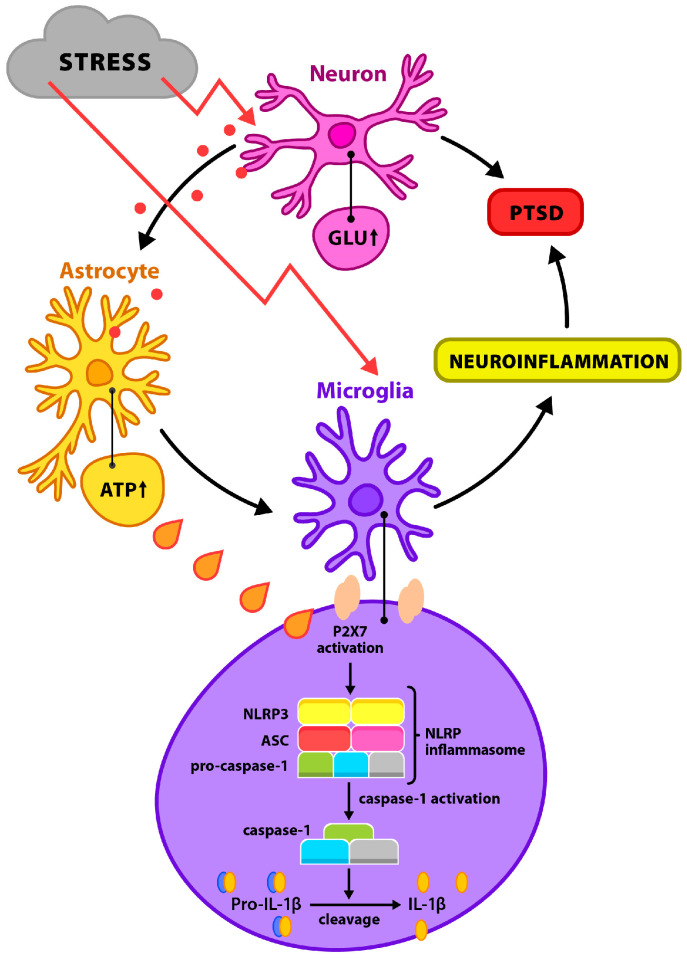
Potential pathological mechanisms of chronic stress.

Chronic stress is a significant factor disrupting the body’s neuroimmune homeostasis, ultimately leading to the development of inflammatory states within the central nervous system (CNS) [1]. The stress response plays an adaptive role by mobilizing survival mechanisms. However, prolonged exposure to stressors initiates molecular and cellular cascades that cause lasting neurobiological changes, increasing vulnerability to mental disorders such as depression, Post-Traumatic Stress Disorder (PTSD), and anxiety disorders [2,3]. A central element of the stress response is the hypothalamic–pituitary–adrenal (HPA) axis, whose activation results in elevated levels of glucocorticoids and adrenocorticotropic hormone. Glucocorticoids, through interaction with Mineralocorticoid Receptors (MR) and Glucocorticoid Receptors (GR) in limbic structures, modulate the expression of genes involved in regulating immune response, neuroplasticity, and energy metabolism [4,5]. Simultaneously, activation of the Sympathetic Nervous System (SNS) leads to the release of catecholamines—noradrenaline and adrenaline—which intensify both peripheral and central inflammatory responses. A key mechanism involves the increased production of pro-inflammatory cytokines such as Interleukin-1β (IL-1β), Interleukin-6 (IL-6), and Tumor Necrosis Factor-α (TNF-α). These cytokines can cross the Blood–Brain Barrier (BBB) or be produced locally by glial cells, leading to a neuroinflammatory phenotype that promotes synaptic dysfunction and neurodegeneration [6,7]. These processes involve the activation of caspase-1 and cytoskeletal changes within the cell, as well as the formation of 1–2 nm pores in the cytoplasmic membrane, enabling the passage of pro-inflammatory cytokines. Additionally, cellular swelling and rupture may occur due to disruptions in osmotic balance. Caspase-1 activation is crucial for the development and maintenance of neuroinflammation through the production and release of IL-1β and Interleukin-18 (IL-18) [8]. Interleukin-1β promotes leukocyte migration and activates a cascade of cytokines and chemokines that sustain inflammation, while IL-18 stimulates T cells and macrophages to produce Interferon Gamma (IFN-γ), Interleukin-1α (IL-1α), IL-6, and TNF-α, further sustaining local inflammation [9,10]. A key player in the neuroinflammatory response to stress is the NOD-Like Receptor Pyrin Domain-Containing Protein 3 (NLRP3) inflammasome (Nucleotide-Binding Domain, Leucine-rich Repeat-Containing Receptor Family, Pyrin Domain-Containing-3)—a cytoplasmic signaling complex that detects Damage-Associated Molecular Patterns (DAMPs) [11]. Once activated, the NLRP3 inflammasome initiates a cascade leading to the activation of caspase-1, which results in the maturation and secretion of pro-inflammatory cytokines IL-1β and IL-18. Excessive NLRP3 activation is associated with neurotoxicity, loss of synaptic integrity, mitochondrial damage, and increased oxidative stress—especially in brain structures vulnerable to stress, such as the hippocampus, amygdala, and prefrontal cortex [12,13]. It is worth noting that the expression and function of the NLRP3 inflammasome are present not only in microglia but also in neurons and astrocytes, which broadens its role in neuroinflammatory pathomechanisms. Chronic activation of this complex promotes disturbances in glutamatergic transmission and induces neuronal apoptosis, contributing to the development of persistent neurobiological changes characteristic of mood and anxiety disorders [14,15], [Figure 1].

In PTSD, stress induces excessive glutamate (Glu) release at neuronal synapses, stimulating astrocytes to release large amounts of extracellular Adenosine Triphosphate (ATP). In microglia, ATP activates P2X7 receptors, triggering a cascade that leads to the overactivation of NLRP3 inflammasomes. This involves the oligomerization of NLRP3, ASC (apoptosis-associated speck-like protein), and pro-caspase-1 into a multiprotein complex. The conversion of pro-caspase-1 into active caspase-1 allows for the cleavage of pro-IL-1β into mature IL-1β, which is secreted and promotes neuroinflammation. Chronic inflammation may disrupt neuronal homeostasis and contribute to the development of depression and PTSD-related symptoms.

The aim of this paper is to present the molecular and cellular mechanisms that disrupt neuroimmune balance in response to chronic stress. It discusses mechanisms regulating the activation of the HPA axis, modulation of glucocorticoid receptors, and cytokine signaling. Particular attention is given to the NLRP3 inflammasome as a central integrator of stress signals that, by modulating glial and neuronal activity, contributes to the development of a neuroinflammatory phenotype and stress-related neuropsychiatric pathologies. The molecular structure of NLRP3 and its role in the neuroinflammatory cascade are presented, along with potential pharmacological strategies for modulating the activity of this complex in the context of treating mental disorders associated with chronic stress.

## 2. Neuroendocrine and Neuroimmune Mechanisms in the Pathophysiology of Stress-Related Disorders

Stress-related psychiatric conditions, including PTSD, depression, and anxiety, stem from intricate interactions between the neuroendocrine and neuroimmune systems. Prolonged stress disrupts HPA axis activity and initiates inflammatory responses within the central nervous system, affecting both neurons and glial cells. Section 2.1 explores HPA axis regulation and outlines how sustained stress drives neuroimmunological imbalance, undermining the body’s ability to preserve homeostasis. Section 2.2 examines the NLRP3 inflammasome as a molecular bridge connecting stress to neuroinflammation, detailing its activation mechanisms, its interplay with the HPA axis, and its potential as a therapeutic target.

### 2.1. The Dynamics of the HPA Axis and Mechanisms of Neuroimmunological Dysregulation in Response to Stress

#### 2.1.1. Dynamics of the HPA Axis in the Stress Response

Stress is a significant factor disrupting the body’s homeostasis and has a considerable impact on the functioning of the CNS [16,17,18]. A growing body of evidence indicates that the stress response is not limited to the activation of the HPA axis but also involves complex neuroinflammatory mechanisms, including microglial activation, inflammasome induction, increased expression of pro-inflammatory cytokines, and destabilization of neuronal networks regulating emotions [19,20,21].

The primary adaptive mechanism in response to stress is the activation of the HPA axis, which leads to the release of Corticotropin-Releasing Hormone (CRH), Adrenocorticotropic Hormone (ACTH), and glucocorticoids—primarily cortisol. Simultaneously, the activation of the sympathetic nervous system results in elevated levels of catecholamines—adrenaline and noradrenaline. Under chronic allostatic load, glucocorticoid resistance develops, impairing mechanisms that suppress neuroinflammatory processes. Dysfunction of the HPA axis contributes to sustained elevated levels of pro-inflammatory cytokines, which in turn affect neurofunctional impairments within the limbic system [22].

Stress signals are transmitted to the CNS through two main pathways: the neural route, via vagus nerve fibers, and the humoral route, involving the passage of pro-inflammatory cytokines into the brain through active transporters or direct action on areas lacking a blood–brain barrier [23]. Pro-inflammatory cytokines, acting on receptors found on astrocytes, microglia, and endothelial cells, activate transcriptional cascades, including Nuclear Factor kappa-light-chain-enhancer of activated B cells (NF-κB) and Mitogen-Activated Protein Kinase (MAPK). In addition, endogenous danger molecules (DAMPs), such as ATP and High Mobility Group Box 1 (HMGB1), stimulate Toll-Like Receptors (TLRs) and components of the NLRP3 inflammasome [24,25,26,27].

A central element of the neuroinflammatory response is the activation of microglia, particularly in brain regions involved in emotion and anxiety regulation—such as the amygdala, hippocampus, prefrontal cortex, and the Bed Nucleus of the Stria Terminalis (BNST). During neuroinflammation, microglia release pro-inflammatory cytokines like IL-6, IL-1β, and TNF-α, which negatively affect cognitive functions, synaptic plasticity, and neurogenesis [28,29].

Chronic activation of the HPA axis leads to neuroplastic and molecular changes in limbic and cortical structures, especially in areas involved in emotional regulation, stress processing, and cognitive control. At the molecular level, hypercortisolemia causes dysregulation of the expression of factors such as Brain-Derived Neurotrophic Factor (BDNF), MAPK, cAMP Response Element-Binding Protein (CREB), and N-Methyl-D-Aspartate (NMDA) and α-Amino-3-hydroxy-5-methyl-4-Isoxazolepropionic Acid (AMPA) receptors, which are essential for neuroplasticity and synaptic transmission. In the hippocampus, pro-inflammatory microglial cytokines impair synaptic plasticity and reduce the proliferation of neuronal precursors in the subgranular zone of the dentate gyrus. IL-1β inhibits Calcium/Calmodulin-Dependent Protein Kinase II (CaM-KII) kinase phosphorylation and NMDA receptor function, leading to impaired memory consolidation [30,31].

In the prefrontal cortex, chronic stress results in dendritic atrophy and a reduction in the number of synaptic spines on layer V pyramidal neurons, which correlates with deficits in executive function and emotion regulation. Cytokines IL-1β and IL-18 promote neuronal hyperreactivity in the basolateral and central amygdala, enhancing fear and anticipatory anxiety responses and hindering the extinction of fear memory. These phenomena may contribute to neurotoxicity and increase the risk of developing affective disorders [32,33,34].

The BNST plays a key role in stress and anxiety regulation. It is functionally connected with the Paraventricular Nucleus (PVN) of the hypothalamus and the amygdala and is involved in the long-term processing of stressors. Caspase-1, activated via autoproteolysis, cleaves pro-interleukins and initiates the formation of membrane pores by the N-terminal of Gasdermin D (GSDMD) protein, leading to cytokine secretion and the induction of pyroptosis—a pro-inflammatory form of cell death [35].

Elevated IL-1β levels in the hippocampus correlate with impaired long-term potentiation (LTP), weakened neurogenesis, and memory consolidation disorders, corresponding to clinical symptoms of depression, cognitive deficits in PTSD, and Alzheimer’s disease [36]. Increased IL-18 concentrations in limbic structures such as the amygdala and hypothalamus are associated with intensified anxiety and depressive responses. IL-1β enhances presynaptic glutamate release while reducing its uptake by astrocytic transporters Glutamate Transporter-1 (GLT-1), leading to excessive NMDA receptor stimulation and excitotoxicity [37].

Activation of the NLRP3 inflammasome also negatively affects the integrity of the BBB. Both IL-1β and IL-18 modulate the expression of adhesion molecules (Intercellular Adhesion Molecule 1—ICAM-1, Vascular Cell Adhesion Molecule 1—VCAM-1) and increase endothelial permeability, resulting in the destabilization of tight junctions and facilitating leukocyte migration into the CNS, thereby intensifying neuroinflammatory processes. The chronic persistence of neuroinflammation stabilizes the inflammatory microglial phenotype, exacerbating limbic structure dysfunction and creating a pathological positive feedback loop in which stress and inflammation reinforce each other. This phenomenon promotes the development of chronic neuropsychiatric disorders such as depression, anxiety, and cognitive impairments [38,39,40].

Brain structures involved in HPA axis function include the PVN of the hypothalamus—the source of CRH, the anterior pituitary which releases ACTH, and the adrenal cortex, where ACTH activates the Melanocortin-2 Receptor (MC2R), initiating glucocorticoid synthesis. The HPA axis is the primary regulator of the stress response, integrating neurohormonal and immune signals that determine adaptation and survival [41].

Glucocorticoids act on CNS cells via MR, NR3C2 and GR, NR3C1. The HPA axis is regulated through negative feedback mechanisms—increased glucocorticoid levels inhibit CRH and ACTH production, preventing HPA overactivation. However, chronic stress impairs this feedback loop, resulting in persistent hypercortisolemia and an intensified glucocorticoid effect on the hippocampus, prefrontal cortex, and amygdala [42,43].

At the molecular level, glucocorticoids modulate neuroinflammatory processes by influencing the transcription of NF-κB and Activator Protein-1 (AP-1), key regulators of pro-inflammatory gene expression. Prolonged glucocorticoid exposure can lead to immune cell resistance, increasing pro-inflammatory cytokine production and exacerbating neuroinflammation [44]. Microglia, as central modulators of the inflammatory response, alter their function in response to HPA signals, reflected in elevated IL-1β and IL-18 production linked to NLRP3 inflammasome activation.

#### 2.1.2. Neuroimmunological Dysregulation and Microglial–Inflammasome Pathways

This inflammasome initiates the neuroinflammatory response via caspase-1 activation and conversion of IL-1β and IL-18 precursors into their active forms [45].

Neuroinflammatory mechanisms, integrating oxidative stress, calcium ion homeostasis disruption, and mitochondrial dysfunction in microglia, lead to destabilization of neuroendocrine balance. The HPA axis serves as the primary regulator of the stress response, integrating hormonal and immune signals essential for adaptation and survival. Its interaction with neuroinflammatory mechanisms—particularly in microglia—is a key pathophysiological mechanism underlying stress-related neuropsychiatric disorders, highlighting the HPA axis’s therapeutic potential in the neuroimmunology of stress [46,47,48].

To summarize, the stress response involves a complex interaction between the nervous, neuroendocrine, and immune systems, which, under chronic stress, leads to the dysregulation of these systems. Activation of the HPA axis, microglia, and the NLRP3 inflammasome initiates a neuroinflammatory cascade which, if persistent, contributes to the development and consolidation of neuropsychiatric disorders.

The next section of this paper will discuss the mechanisms by which the NLRP3 inflammasome influences CNS pathophysiology.

### 2.2. The NLRP3 Inflammasome as a Molecular Mediator of the Neuroinflammatory Response to Stress: Activation Mechanisms, Interaction with the HPA Axis, and Therapeutic Implications

The NLRP3 inflammasome serves as a pivotal cellular sensor connecting stress to brain inflammatory responses. Once activated, it promotes the release of proinflammatory cytokines (IL-1β, IL-18), impacting neuronal and glial function and potentially contributing to conditions such as PTSD and depression. Section 2.2.1 examines the mechanisms driving NLRP3 activation and its role in intensifying neuroinflammation within the central nervous system. Section 2.2.2 addresses the interaction between NLRP3 and the HPA axis, emphasizing how these links may be leveraged for therapeutic intervention.

#### 2.2.1. Activation Mechanisms of the NLRP3 Inflammasome and Its Role in Modulating the Neuroinflammatory Response

The NLRP3 inflammasome (NOD-like receptor pyrin domain-containing 3) is one of the best-characterized immune signaling complexes, whose activation leads to the production of pro-inflammatory cytokines and the initiation of pyroptosis. Its role in the neurobiology of stress has gained particular attention in recent years, as numerous preclinical and translational studies have shown that the activation of this complex contributes to the emergence and persistence of neuroimmune dysfunctions typical of affective disorders, depression, post-traumatic stress disorder, and anxiety disorders [49].

NLRP3 belongs to the family of cytoplasmic Pattern Recognition Receptors (PRRs), which act as molecular danger signals. Structurally, this protein consists of three main domains: the Leucine-Rich Repeat (LRR) domain, which participates in danger signal detection and maintains the protein in an inactive state; the NACHT domain, responsible for oligomerization; and the Pyrin Domain (PYD), which is involved in recruiting the adaptor protein Apoptosis-Associated Speck-Like Protein Containing a Caspase Activation and Recruitment Domain (ASC a CARD) through PYD–PYD homotypic interactions [50,51].

**Figure 2:**
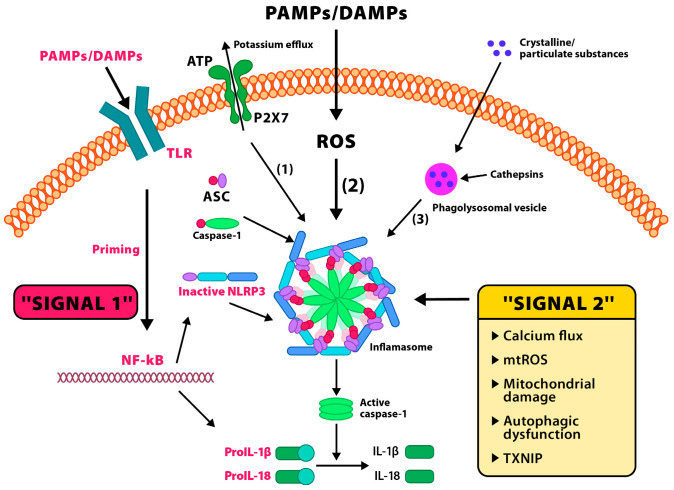
Two-step activation of the NLRP3 inflammasome.

Inflammasome activation occurs in two phases [Figure 2].

Activation of the NLRP3 inflammasome occurs in two steps. Signal 1 (priming): TLRs recognize pathogen-associated molecular patterns (PAMPs) or DAMPs, leading to the activation of the transcription factor NF-κB. In the nucleus, NF-κB promotes the transcription of NLRP3, pro-IL-1β, and pro-IL-18 genes. The translated proteins remain in the cytoplasm in their inactive forms. Signal 2 (activation): A secondary stimulus leads to the assembly of the inflammasome complex composed of NLRP3, the adaptor protein ASC, and pro-caspase-1. This complex facilitates the conversion of pro-caspase-1 into its active form, caspase-1, which in turn cleaves pro-IL-1β and pro-IL-18 into their mature, secreted proinflammatory forms, IL-1β and IL-18. Three major mechanisms of signal 2 activation: ATP activates the P2X7 receptor, causing K^+^ efflux and Ca^2+^ influx—critical for inflammasome activation; reactive oxygen species (ROS) promote NLRP3 complex oligomerization; lysosomal rupture (e.g., after phagocytosis of crystals) releases enzymes such as cathepsin B, which also activate the inflammasome. Additional contributing factors include mitochondrial damage, autophagic dysfunction, and the involvement of thioredoxin-interacting protein (TXNIP).

The first phase—priming—involves transcriptional upregulation of NLRP3, pro-IL-1β, and pro-IL-18 through the activation of the TLR4 or proinflammatory cytokine receptors that stimulate the NF-κB pathway. In the second—activation—phase, various molecular signals such as K^+^ efflux, increased Ca^2+^ concentration, mitochondrial DNA (mtDNA), ROS production, crystalline structures (e.g., uric acid crystals), or TXNIP translocation lead to conformational changes in NLRP3, its oligomerization, and the formation of an active complex with ASC and procaspase-1 [52]. A critical element in NLRP3 inflammasome activation in mammalian cells is NIMA-Related Kinase 7 (NEK7), which binds to the NACHT domain of NLRP3, stabilizing the complex structure and enabling its functional activation. In vitro studies have shown that deletion of NEK7 prevents procaspase-1 recruitment and IL-1β production in response to inflammasome-activating signals, making this kinase a key therapeutic target in neuroinflammatory conditions [52]. In the central nervous system, the primary reservoirs of the NLRP3 inflammasome are microglia and astrocytes [53,54]. In response to stressors (such as chronic psychosocial stress, sleep deprivation, or metabolic stress), microglia transition from a homeostatic phenotype to a “primed” state, characterized by a lowered activation threshold, increased production of cytokines (IL-1β, IL-18), and decreased expression of neuroprotective proteins such as Triggering Receptor Expressed on Myeloid Cells 2 (TREM2) and CX3C Chemokine Receptor 1 (CX3CR1). In this primed state, even minimal exposure to stress stimuli can lead to full inflammasome activation [55].

Preclinical studies using rodent models (e.g., Chronic Restraint Stress—CRS) have shown that stress activates microglia and increases the expression of NLRP3 mRNA and caspase-1 in the hippocampus, paraventricular nucleus, and amygdala [56,57]. In PTSD models (e.g., single prolonged stress), elevated IL-1β levels and increased microglial pyroptosis (detection of N-terminal of Gasdermin D—GSDMD-N) have been observed [58]. These effects were reversed by pharmacological inhibition of the inflammasome (MCC950), which improved exploratory behavior and reduced anxiety- and depression-like symptoms. In neurons, chronic stress and its associated neurotoxicity can lead to NLRP3 expression. The cytokines IL-1β and IL-18 affect synaptic transmission by inhibiting LTP, reducing dendritic spine density, and lowering BDNF levels, contributing to impaired neuroplasticity, memory, and learning [59]. Given the key role of the NLRP3 inflammasome in the pathogenesis of neuropsychiatric disorders such as treatment-resistant depression, PTSD, bipolar disorder, or schizophrenia with an inflammatory component, its pharmacological and non-pharmacological inhibition represent a promising direction for targeted molecular therapy [60]. This inflammasome, through the activation of caspase-1 and maturation of proinflammatory cytokines IL-1β and IL-18, plays a central role in perpetuating neuroinflammatory cascades leading to neurotransmitter dysregulation, blood–brain barrier disruption, and impaired neuroplasticity [61]. Therefore, interventions aimed at suppressing its activity may restore immunoneuronal balance and improve clinical outcomes in psychiatric disorders. One of the most promising therapeutic approaches is the use of selective NLRP3 inflammasome inhibitors, the best-known of which is MCC950 (also known as Cytokine Release Inhibitory Drug 3—CRID3) [62]. This compound has high specificity for the NACHT domain of NLRP3, preventing its oligomerization and caspase-1 activation. In numerous animal models, MCC950 has been shown to reduce IL-1β and IL-18 levels in the hippocampus and amygdala, decrease microgliosis, and reverse anhedonia, depressive, and anxiety-like behaviors [63,64]. Importantly, MCC950 treatment did not negatively affect physiological immune responses, making it a safer alternative to non-selective cytokine inhibitors [65]. Another important anti-inflammatory and inflammasome-modulating agent is metformin—a commonly used type 2 diabetes drug. Metformin exerts neuroprotective effects by activating AMP-Activated Protein Kinase (AMPK), which regulates cellular energy homeostasis and inhibits the production of mitochondrial reactive oxygen species (mtROS), a key trigger for NLRP3 activation [66]. In vivo studies have shown that metformin administration reduces NLRP3 and caspase-1 expression in the hippocampus of chronically stressed mice, contributing to improved neurogenesis and cognitive function. MCC950, a selective small-molecule inhibitor of the NLRP3 inflammasome, exhibits high specificity for the NACHT domain, blocking ATP hydrolysis, oligomerization, and subsequent caspase-1 activation. Preclinical research demonstrates that MCC950 can cross the BBB via intraperitoneal (i.p.) or intranasal administration, lowering IL-1β and IL-18 concentrations in the hippocampus and amygdala and improving behavioral outcomes in chronic stress and PTSD models. Metformin, despite its hydrophilic nature, has been shown to cross the BBB through organic cation transporters (OCTs) after intraperitoneal or oral administration, supporting its potential for clinical translation [67]. β-hydroxybutyrate (β-HB), an endogenous ketone body, readily crosses the BBB via monocarboxylate transporters (MCT1/2) and can be administered orally, intraperitoneally, or intranasally. Intranasal β-HB provides rapid CNS entry and has been shown to reduce microglial activation and restore synaptic plasticity in stress-related models. Owing to their BBB permeability and demonstrated ability to modulate neuroinflammation, MCC950, metformin, and β-HB represent strong translational candidates, with intranasal delivery offering a particularly promising approach to maximize CNS bioavailability while minimizing systemic effects [68]. This effect was particularly pronounced in animals with induced insulin resistance, emphasizing the importance of the metabolism–inflammation axis in neuropsychiatry [69]. Another interesting compound is β-HB, a natural ketone produced endogenously during fasting, ketogenic dieting, or intense physical activity. β-HB inhibits the NLRP3 inflammasome by stabilizing mitochondrial membrane potential, reducing mtROS levels, and disrupting interactions between NLRP3 and NEK7—a key molecule necessary for inflammasome activation. Supplementation with β-HB in stress-induced depression models led to reduced anxiety-like behaviors and restored normal dendritic spine morphology in the prefrontal cortex [70]. Minocycline, an antibiotic from the tetracycline group, is known not only for its antimicrobial properties but also for its strong immunomodulatory effects in the central nervous system. It inhibits microglial activation, reduces the secretion of inflammatory cytokines such as Tumor Necrosis Factor Alpha (TNF-α) and IL-6, and limits the expression of ASC and NLRP3 proteins. In both clinical and preclinical studies, minocycline has shown beneficial effects in the treatment of depression, particularly in individuals with elevated serum inflammatory markers (e.g., CRP, IL-6). Its antidepressant and anti-inflammatory effects were correlated with reduced amygdala volume and improved executive function [71]. Alongside pharmacological interventions, there is growing interest in non-pharmacological strategies targeting the gut–brain axis. Chronic psychological stress leads to gut microbiota dysbiosis, loss of immunotolerant strains (such as *Faecalibacterium prausnitzii* or *Bifidobacterium infantis*), and increased intestinal permeability. This promotes the translocation of PAMPs, (e.g., Lipopolysaccharide—LPS) into systemic circulation, which can activate the NLRP3 inflammasome via TLR receptors in microglial cells. Supplementation with anti-inflammatory probiotics (e.g., *Lactobacillus rhamnosus* GG, *Bifidobacterium breve*) and a fiber-rich, fermentable diet promoting the production of short-chain fatty acids such as butyrate may reduce inflammasome activity, improve blood–brain barrier integrity, and modulate HPA axis function [72].

#### 2.2.2. Interactions Between the NLRP3 Inflammasome and HPA Axis Regulation: Opportunities for Therapeutic Modulation

The HPA axis is the principal neuroendocrine pathway coordinating the body’s stress response and maintaining homeostasis under threat. Its activation induces the release of CRH from the hypothalamus, ACTH from the pituitary, and glucocorticoids—cortisol in humans and corticosterone in rodents—from the adrenal cortex. In acute stress, glucocorticoids exert strong immunosuppressive effects by suppressing proinflammatory gene expression and limiting immune cell activation, including components of the NLRP3 inflammasome. This is achieved via glucocorticoid binding to the GR, nuclear translocation of the GR–ligand complex, and recruitment of transcriptional corepressors such as histone deacetylase 2 (HDAC2), which collectively inhibit NF-κB activation. As NF-κB is a central transcription factor for NLRP3, pro–IL-1β, and pro–IL-18, its suppression effectively reduces inflammasome priming [73].

Chronic HPA axis activation, as seen in persistent psychosocial stress, PTSD, or treatment-resistant depression, results in glucocorticoid resistance—marked by diminished GR sensitivity in immune cells, including microglia—which weakens glucocorticoid-mediated suppression of inflammatory gene expression. In preclinical stress models, prolonged corticosterone exposure increases microglial vulnerability to inflammasome activation, elevating NLRP3, IL-1β, and caspase-1 transcription. Mechanistically, this involves disruption of the GR–HDAC2 pathway, overexpression of TXNIP, and increased mitochondrial membrane permeability to mtDNA, which serves as a direct inflammasome-activating signal [74,75].

Chronic stress also perturbs gut microbiota composition, an important regulator of neuroimmune balance. Stress-related dysbiosis features reduced abundance of immunotolerant species such as *Faecalibacterium prausnitzii* and *Bifidobacterium infantis*, along with increased intestinal permeability (“leaky gut”), enabling PAMPs such as LPS to enter systemic circulation. LPS activates TLR4 on microglia and monocytes, initiating the priming phase of NLRP3 activation by inducing transcription of NLRP3 and pro–IL-1β. Simultaneously, a decline in butyrate—a short-chain fatty acid with neuroprotective and anti-inflammatory properties—enhances TXNIP expression and mtROS generation, both of which act as secondary inflammasome-activating signals [76].

Evidence for microbiota-driven modulation of the HPA–NLRP3 axis comes from studies where microbiota from chronically stressed donors were transplanted into germ-free recipients. Even without direct stress exposure, these recipients exhibited elevated NLRP3 expression, increased hippocampal microgliosis, and behavioral signs of anhedonia and avoidance [77,78], indicating that dysbiosis alone can sustain microglial priming and inflammasome activity.

Clinically, persistent HPA axis hyperactivation and NLRP3 inflammasome signaling contribute to neuroinflammatory pathology across multiple psychiatric disorders [15,38,40,43,44]. In PTSD, chronic HPA dysregulation is associated with sustained neuroimmune activation and elevated CNS IL-1β levels. In treatment-resistant depression and bipolar disorder, ongoing inflammasome activity impairs synaptic plasticity, reduces BDNF expression, and hinders hippocampal neurogenesis. In inflammatory-subtype schizophrenia, similar mechanisms occur, including blood–brain barrier breakdown, vascular hyperpermeability, and infiltration of peripheral immune cells into brain tissue [37,61].

Experimental studies further clarify how stress-induced NLRP3 activation influences neuron–glia communication and behavior. Dong et al. [79] found that psychological stress activates NLRP3 in microglia, increasing IL-1β release and impairing fear memory extinction in mice, an effect reversed by MCC950. Yang et al. [80] reported that in a PTSD rat model, chronic stress not only activated NLRP3 but also disrupted hippocampal myelin, linking inflammasome signaling to white matter integrity. Ji et al. [81] identified astrocytic NLRP3 as a driver of pathological fear memory, showing that leptin inhibited astrocytic NLRP3 activation, reduced IL-1β release, and alleviated PTSD-like symptoms. Grzesińska and Ogłodek [82] described the interaction between NLRP3, matrix metalloproteinases (MMP-2, MMP-9), and GABAergic signaling in PTSD, suggesting that inflammasome-driven extracellular matrix remodeling and impaired inhibitory neurotransmission may synergistically worsen neuroinflammation and cognitive–emotional dysfunction.

In the healthy brain, microglia act as highly specialized environmental sensors, continuously monitoring synaptic activity, eliminating redundant connections through synaptic pruning, and supporting synaptic plasticity and neurogenesis. Astrocytes, in turn, maintain ionic homeostasis, regulate neurotransmitter metabolism—including glutamate uptake via EAAT1/2 transporters—supply neurons with metabolic substrates through the astrocyte–neuron lactate shuttle, and sustain the integrity of the BBB [83]. Under chronic stress conditions, profound phenotypic changes occur in glial cells. Microglia shift toward a pro-inflammatory M1 phenotype, characterized by increased production of pro-inflammatory cytokines such as IL-1β, IL-6, and TNF-α, as well as chemokines (e.g., CX3CL1 and CCL2). Concurrently, astrocytes undergo reactive transformation (astrogliosis), losing part of their neuroprotective properties and increasing the release of inflammatory mediators, including ATP, nitric oxide, and the S100B protein, which further amplifies the neuroinflammatory milieu [84]. The NLRP3 inflammasome, localized predominantly in microglia, is a key regulator of neuroinflammation induced by chronic stress [85]. Its activation proceeds in two stages. In the priming phase, the inflammatory response is initiated by neuron-derived DAMPs such as ATP released during excessive membrane depolarization, heat shock proteins, or mitochondrial DNA fragments. These ligands bind to PRRs, such as TLR2, TLR4, or NOD-like receptors, on microglia and astrocytes, activating the NF-κB pathway and upregulating the transcription of NLRP3 and pro-IL-1β. In the activation phase, secondary signals are required, including mitochondrial membrane depolarization, potassium efflux, calcium influx, excessive production of mtROS, and accumulation of cholesterol or amyloid-β crystals. Full inflammasome activation leads to the recruitment of the adaptor protein ASC and pro-caspase-1, which undergoes autoproteolysis to form active caspase-1 [6,8,11]. This protease catalyzes the maturation of pro-IL-1β and pro-IL-18 into their active forms, which are secreted into the extracellular space. NLRP3 activation profoundly alters communication between neurons and glial cells. In microglia–neuron interactions, IL-1β acts on IL-1R1 receptors on neurons, modulating kinase activity and influencing the phosphorylation of NMDA and AMPA glutamatergic receptors. This increases Ca^2+^ influx, exacerbates excitotoxicity, and leads to dendritic spine loss. IL-18 further reduces BDNF expression, impairing hippocampal neurogenesis [15,25,26,27,37]. In microglia–astrocyte interactions, cytokines and signaling molecules released from activated microglia induce a reactive A1 astrocyte phenotype, marked by diminished glutamate uptake and inhibition of neurotrophic factor production, while increasing the secretion of neurotoxic mediators such as NO, ROS, and extracellular matrix proteases. Reactive astrocytes, in turn, disrupt astrocyte–neuron homeostasis by excessively releasing glutamate into the synaptic cleft and limiting the supply of metabolic substrates, including lactate, thereby aggravating neuronal excitotoxicity and metabolic deficits [40,43,50].

Clinically, sustained NLRP3 activation under chronic psychological stress disrupts neuron–microglia–astrocyte networks, leading to persistent synaptic dysfunction, reduced neuronal plasticity, and impaired neurogenesis. These alterations manifest as long-term cognitive deficits, emotional dysregulation, and heightened relapse risk in PTSD, depression, and anxiety disorders [53,54,55].

Given these converging lines of evidence, pharmacological targeting of the NLRP3 inflammasome represents a promising avenue for therapeutic intervention in neuropsychiatric disorders with an inflammatory basis. Selective inhibitors such as MCC950 block NACHT domain oligomerization, while metabolic and mitochondrial modulators, including metformin (an AMPK activator) and β-hydroxybutyrate (a ketone body that disrupts NLRP3–NEK7 binding), provide complementary effects by reducing oxidative stress and restoring mitochondrial stability. In parallel, non-pharmacological approaches—such as microbiota-targeted interventions with probiotics, prebiotics, and fermentable fiber diets—can restore SCFA production, strengthen gut and blood–brain barrier integrity, and normalize stress responses (Table 1 summarizes selected NLRP3 inhibitors, their molecular targets, and evidence from preclinical and clinical studies) [72,77].

Despite strong preclinical efficacy, translation of these inhibitors into clinical use is hindered by significant challenges. Pharmacokinetic and pharmacodynamic differences between species affect optimal dosing and bioavailability, while the multifactorial nature of human psychiatric disorders cannot be fully replicated in CRS or single prolonged stress (SPS) models. Moreover, the absence of validated in vivo biomarkers for NLRP3 activity hampers patient selection and treatment monitoring, and the long-term safety of chronic inflammasome inhibition remains uncertain, particularly in relation to host defense against infection [27,37,40]. Additionally, compensatory activation of alternative inflammatory pathways may limit therapeutic benefit.

Accordingly, developing clinically viable biomarkers is a priority. Potential candidates include circulating or cerebrospinal fluid (CSF) concentrations of IL-1β, IL-18, and ASC protein; NLRP3 expression in peripheral monocytes; gut microbiota-derived metabolite profiles—particularly short-chain fatty acids (SCFAs) such as butyrate; plasma mtDNA levels; and neuroimaging indicators such as PET ligands for microglial activation (e.g., TSPO) or functional MRI connectivity patterns. These measures could facilitate personalized interventions targeting the HPA–NLRP3 axis, enabling the identification of likely responders and the real-time monitoring of therapeutic effects [43,53,86,87].

An effective treatment paradigm will likely require an integrative strategy that combines pharmacological NLRP3 inhibition with approaches to restore microbiota balance, support mitochondrial function, and reduce stress reactivity [26,50]. Multimodal interventions—such as MCC950 or metformin administration, probiotic supplementation, dietary adjustments, cognitive–behavioral therapy, mindfulness practices, and moderate aerobic exercise—may improve long-term efficacy and reduce relapse risk in stress-related neuropsychiatric conditions.

## 3. Conclusions

Activation of the NLRP3 inflammasome is a key component of the neuroinflammatory response to chronic stress, integrating endocrine and metabolic signals with microglial and astrocytic responses. This process leads to increased production of proinflammatory cytokines, impaired synaptic plasticity, and neuronal damage in stress-sensitive brain regions. Chronic inflammasome activation promotes the development of neuropsychiatric disorders such as depression and PTSD. Targeting the NLRP3 inflammasome represents a promising therapeutic strategy. Selective inhibitors and immunomodulation—both pharmacological and non-pharmacological—can help restore neuroimmune homeostasis. The introduction of personalized therapies will require further research into the molecular mechanisms and biomarkers of inflammasome activation.

## Figures and Tables

**Table 1:** Selected NLRP3 inflammasome inhibitors: molecular targets, preclinical and clinical evidence.

Inhibitor	Molecular Target/Mechanism	Preclinical Evidence	Clinical Evidence/Translational Notes
MCC950 (CRID3)	Binds to NACHT domain of NLRP3, blocks ATP hydrolysis and oligomerization	Reduces IL-1β/IL-18 in hippocampus and amygdala, reverses depressive/anxiety-like behaviors in CRS and SPS models	No approved human use; Phase I safety data limited; hepatotoxicity concerns require further study
Metformin	Activates AMPK, reduces mtROS, inhibits TXNIP–NLRP3 interaction	Improves neurogenesis, reduces NLRP3/caspase-1 in hippocampus of stressed mice; benefits in metabolic-inflammation comorbidity	Approved T2DM drug; epidemiological links to reduced depression risk; no targeted NLRP3 trials
β-HB	Stabilizes mitochondrial membrane potential, inhibits NEK7–NLRP3 interaction	Restores dendritic spine morphology, reduces anxiety-like behavior in stress-induced depression models	Endogenous ketone; ketogenic diets tested in epilepsy and mood disorders; no direct NLRP3-focused trials
Minocycline	Inhibits microglial activation, reduces ASC/NLRP3 expression, lowers TNF-α and IL-6	Attenuates neuroinflammation, improves cognition and mood in rodent stress models	Small clinical trials show antidepressant effect in high-CRP depression; broader trials needed
OLT1177 (Dapansutrile)	Selective NLRP3 inhibitor; prevents ASC speck formation	Cardiovascular and neuroprotective benefits in preclinical ischemia models	Phase II in gout and heart failure; potential repurposing for neuropsychiatric inflammation
CY-09	Blocks ATP binding to NLRP3 NACHT domain	Effective in reducing IL-1β release in LPS/ATP-stimulated microglia	No human trials; promising BBB penetration profile in animal models

## Data Availability

All data and analysis are available within the manuscript, or upon request to the corresponding author.

## Abbreviations

The following abbreviations are used in this manuscript:

CNS	Central Nervous System
NLRP3	NOD-Like Receptor Pyrin Domain-Containing Protein 3
HPA axis	Hypothalamic–Pituitary–Adrenal Axis
DAMPs	Damage-Associated Molecular Patterns
IL-1β	Interleukin 1 β
IL-18	Interleukin 18
PTSD	Post-Traumatic Stress Disorder
MR	Mineralocorticoid Receptor
GR	Glucocorticoid Receptor
SNS	Sympathetic Nervous System
IL-6	Interleukin 6
TNF-α	Tumor Necrosis Factor Alpha
IL-1α	Interleukin-1 -α
BBB	Blood–Brain Barrier
IFN-γ	Interferon Gamma
CRH	Corticotropin-Releasing Hormone
ACTH	Adrenocorticotropic Hormone
TLRs	Toll-Like Receptors
NF-κB	Nuclear Factor kappa-light-chain-enhancer of activated B cells
MAPK	Mitogen-Activated Protein Kinase
ATP	Adenosine Triphosphate
BNST	Bed Nucleus of the Stria Terminalis
BDNF	Brain-Derived Neurotrophic Factor
CREB	cAMP Response Element-Binding Protein
NMDA receptor	N-Methyl-D-Aspartate Receptor
AMPA receptor	α-Amino-3-hydroxy-5-methyl-4-isoxazolepropionic acid Receptor
CaM-KII	Calcium/Calmodulin-Dependent Protein Kinase II
LTP	Long-Term Potentiation
GLT-1	Glutamate Transporter 1
ICAM-1	Intercellular Adhesion Molecule 1
VCAM-1	Vascular Cell Adhesion Molecule 1
PVN	Paraventricular Nucleus (of the hypothalamus)
GSDMD	Gasdermin D
MC2R	Melanocortin 2 Receptor
AP-1	Activator Protein 1
ASC	Apoptosis-Associated Speck-Like Protein Containing a CARD
PYD	Pyrin Domain
CARD	Caspase Activation and Recruitment Domain
TLR4	Toll-Like Receptor 4
ROS	Reactive Oxygen Species
TXNIP	Thioredoxin-Interacting Protein
NEK7	NIMA-Related Kinase 7
TREM2	Triggering Receptor Expressed on Myeloid Cells 2
CX3CR1	CX3C Chemokine Receptor 1
CRS	Chronic Restraint Stress
GSDMD-N	N-terminal of Gasdermin D
HDAC2	Histone Deacetylase 2
mtDNA	Mitochondrial DNA
PAMPs	Pathogen-Associated Molecular Patterns
LPS	Lipopolysaccharide
MCC950 (CRID3)	MCC950 (Cytokine Release Inhibitory Drug 3)
AMPK	AMP-Activated Protein Kinase
mtROS	Mitochondrial Reactive Oxygen Species
β-HB	Beta-Hydroxybutyrate
CRP	C-Reactive Protein
TLR	Toll-Like Receptor
HMGB1	High Mobility Group Box 1
PRRs	Pattern Recognition Receptors
LRR	Leucine-Rich Repeat
